# Schizophrenia Associated with Epileptiform Discharges without Seizures Successfully Treated with Levetiracetam

**DOI:** 10.3389/fpsyt.2017.00012

**Published:** 2017-02-08

**Authors:** Dominique Endres, Evgeniy Perlov, Bernd Feige, Dirk-Matthias Altenmüller, Nils Venhoff, Ludger Tebartz van Elst

**Affiliations:** ^1^Section for Experimental Neuropsychiatry, Department of Psychiatry, Faculty of Medicine, University of Freiburg, Freiburg, Germany; ^2^Freiburg Epilepsy Center, Department of Neurosurgery, Faculty of Medicine, University of Freiburg, Freiburg, Germany; ^3^Department of Rheumatology and Clinical Immunology, Faculty of Medicine, University of Freiburg, Freiburg, Germany

**Keywords:** epilepsy, schizophrenia, epileptiform discharges, levetiracetam, paraepileptic, LANI hypothesis

## Abstract

**Background:**

Schizophrenia-like disorders can be divided into endogenic or primary, idiopathic, polygenetic forms, and different secondary, organic subgroups [e.g., (para)epileptic, immunological, degenerative]. Epileptic and paraepileptic explanatory approaches have a long tradition due to the high rate of electroencephalography (EEG) alterations in patients with schizophrenia.

**Case presentation:**

We present the case of a 23-year-old female patient suffering, since the age of 14 years, from a fluctuating paranoid-hallucinatory syndrome with formal thought disorder, fear, delusions of persecution, auditory, visual, and tactile hallucinations, as well as negative and cognitive symptoms. Laboratory measurements showed increased titers of antinuclear antibodies (ANAs) in the context of ulcerative colitis. While there was no clear history or evidence of epileptic seizures, the EEG showed generalized 3 Hz polyspike wave complexes. Under treatment with levetiracetam, the symptoms disappeared and the patient was able to complete vocational training.

**Conclusion:**

The schizophrenia-like symptoms associated with epileptiform discharges but not overt seizures and the good response to antiepileptic treatment could be interpreted in the context of a (para)epileptic pathomechanism. The EEG alterations might be due to a polygenetic effect due to different genes. Mild immunological mechanisms in the framework of ulcerative colitis and increased ANA titers might have supported the network instability. This case report illustrates (1) the importance of EEG screenings in schizophrenia, (2) a potential pathogenetic role of epileptiform discharges in a subgroup of patients with schizophrenia-like symptoms, and (3) that antiepileptic medication with levetiracetam could be a successful treatment alternative in schizophrenia-like disorders with EEG alterations.

## Background

Schizophrenia-like disorders are characterized by delusional perception and delusions of control, hallucinations (e.g., commenting or discussing voices), thought insertion or withdrawal, cognitive impairment, thought disorders, or social withdrawal.^1^ In addition to primary, endogenic or idiopathic, polygenetic forms, different secondary pathophysiological mechanisms [e.g., (para)epileptic, immunological, degenerative] can be assumed. Because of the high rates of electroencephalography (EEG) alterations, ranging from 7 to 60% in patients with schizophreniform syndromes, epileptic and paraepileptic explanatory approaches have a long tradition (1–3). In line with this assumption, we reported the first case of a young patient with a schizophrenia-like disorder, generalized spike-and-slow-wave complexes without epileptic seizures but with remission under treatment with valproate (4, 5). Immunological reasons might be due to autoantibody-associated autoimmune encephalitis, cerebral vasculitis, or collagenosis [e.g., systemic lupus erythematosus (SLE)] (6). Immunological effects might lead to network instability and therefore cause (para)epileptic phenomena (7). The detection of a (para)epileptic or immunological mechanism opens new treatment perspectives, in that antiepileptics or immunomodulators may be helpful (4, 5, 7–10).

## Case Presentation

### Clinical Presentation

We present the case of a 23-year-old female office clerk suffering from fluctuating paranoid-hallucinatory symptoms since the age of 14 years (2007). Therefore, the diagnosis of paranoid schizophrenia was made by different psychiatrists. Although taking neuroleptics, in the course of the disease, the patient developed five episodes (for several weeks) with paranoid-hallucinatory exacerbation. In these episodes, the patient suffered from formal thought disorder, fear, delusions of persecution, auditory hallucinations with commenting, discussing, and commanding voices, visual hallucinations with seeing maggots in her room, and tactile hallucinations with the feeling of being touched from behind. In parallel to these exacerbations, the patient developed severe negative and cognitive symptoms including attention and memory deficits, fatigue, depressive mood, and sleep disturbances thus completing the psychopathological features of comprehensive schizophrenia. Neurological and medical examinations were normal.

### Family History

There was a positive family history for unipolar depression, which was diagnosed earlier in two sisters, both parents, and both grandmothers. There was no history for schizophrenia-like psychopathology, bipolar disorder, or epilepsy.

### Somatic and Developmental History

Symptoms started 6 weeks after pain of the large joints. Therefore, a rheumatological disease was discussed. During an external work-up of repeated diarrhea, a chronic inflammation gut disease (ulcerative colitis) was diagnosed in 2014 and treated with mesalazine. No birth complications or *in utero* abnormalities were remembered; the birth was performed by cesarean section. The early childhood development was normal. No febrile convulsions or inflammatory brain diseases were remembered. The patient suffered mild cerebral contusions at the age of 4 and 12 years.

### Diagnostic Findings

**Figure 1:**
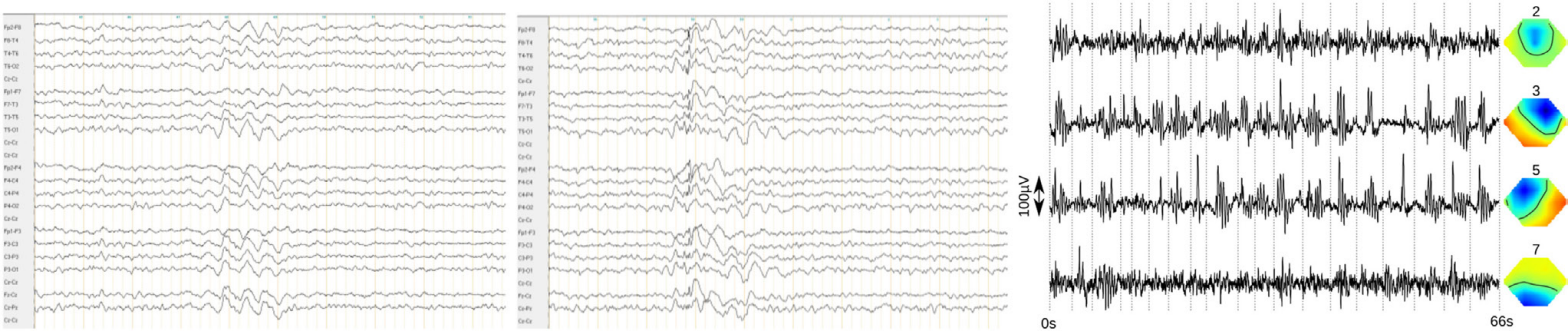
**Frontal accentuated intermittent rhythmic delta activity (FIRDA, left) and generalized 3 Hz polyspike wave complexes (middle) in the bipolar longitudinal rows (7 µV/mm, 0.3 s, 70 Hz)**. **The findings of the independent component analysis are presented in the right picture** [*the following four relevant components were found, left: activity traces, with examples of atypical activity cut from the clinical electroencephalography and appended at the dotted lines. Right: topographies, nose upward, negative (direction opposite of activity trace) blue, positive (direction same as activity trace) red. Right (component 3) and left (component 5) frontal activity show most prominent ~2.6 Hz bursts. Higher frequency activity frontocentral (component 2) and occipital (alpha component 7) are partially related*].

The diagnostic findings are summarized in Table 1. Taken together, the immunological alterations were compatible with the previously known ulcerative colitis (11). The electrophysiological findings (Figure 1) would be compatible with primary (idiopathic) generalized epilepsy; however, the history for epileptic seizures including absences and myoclonic jerks was negative.

**Table 1:** **Diagnostic findings**.

Serum basic diagnostics and blood count	Normal renal, liver, and thyroid values; Slightly increased C3d concentration (11.1 mg/l; reference value <9 mg/l); Normal blood count.
Serum autoantibody analyses	Normal thyroid autoantibodies (*against thyroglobulin, thyroid peroxidase, and thyroid-stimulating hormone*); Rheumatological screening: increased antinuclear antibodies (titer: 1:400; reference value <1:50) without clear extractable nuclear antigens; the anti-nucleosome antibodies were weakly positive; No antibodies against intracellular onconeural antigens (*Yo, Hu, CV2/CRMP5, Ri, Ma1/2, SOX1*), or the intracellular synaptic antigens (*GAD, amphiphysin*).
Cerebrospinal fluid (CSF) analyses	Normal white cell count, no blood–brain barrier dysfunction (normal protein concentration and albumin quotient); No CSF-specific oligoclonal bands, but a weak identical band in the CSF and serum; Antibodies against neuronal cell surface antigens [*NMDAR, AMPA-R, GABA-B-R, VGKC complex* (*LGI1, Caspr2*)] were negative.
Cerebral magnetic resonance imaging (1.5 T)	Normal brain findings; Additional examination findings included a benign lesion of the right frontoparietal skull without contrast enhancement (*most likely equivalent with dermoid cysts; the criteria for monoclonal gammopathy of undetermined significance or multiple myeloma were not fulfilled*).
Electroencephalography (*during the first admission to our clinic in 2013, under the treatment with clozapine, aripiprazole, and citalopram*)	Frontal accentuated intermittent rhythmic delta activity (FIRDA) and generalized 3 Hz polyspike wave complexes.

### Differential Diagnosis

The schizophreniform symptoms fulfilled the criteria of paranoid-hallucinatory schizophrenia (see footnote 1). Therefore, the most important differential diagnoses would be schizophrenia plus coincidental epileptiform discharges. Although the findings of the investigation led us to our consideration of neuropsychiatric SLE, the American College of Rheumatology classification criteria for SLE were not fulfilled.^2^

### Therapy and Outcome

External neuroleptic treatment with risperidone (4 mg), amisulpride (600 mg), perazine (100 mg), aripiprazole (15 mg), and clozapine (275 mg) did not lead to long-term stabilization. Additional treatment with fluoxetine (20 mg) and citalopram (20 mg) for affective, negative, and cognitive symptoms did not successfully improve these symptoms. During the first visit in our clinic, in 2013, we detected the abovementioned epileptiform discharges. Assuming a (para)epileptic pathomechanism, we added antiepileptic treatment with valproate (1,500 mg) to the neuroleptic medication with clozapine and aripiprazole. At this point, the cognitive deficits improved significantly. Also, the EEG improved except for the (F)IRDAs. Another paranoid-hallucinatory episode in 2014 was treated successfully with a dose increase of clozapine and valproate. Because of a strong weight gain, the therapy with valproate was changed to topiramate (200 mg) in 2014. Assuming a (para)epileptic pathomechanism, clozapine was reduced and stopped in 2014. Aripiprazole was reduced in January 2014 and stopped in 2015. Normal results were found in both the routine EEG and in the EEG after sleep deprivation (2014). In the further course, topiramate led to a severe loss of appetite and was therefore changed to levetiracetam (1,500 mg) in 2014. The mental condition stabilized with the short-term antiepileptic treatment with topiramate and the subsequent antiepileptic treatment with levetiracetam (since 2014, and since 2015 as monotherapy). There were no more paranoid-hallucinatory episodes, the negative symptoms declined, the patient became a mother (in the spring of 2016); she lived alone, took care of her daughter, and simultaneously finished her vocational training (in the summer of 2016). She was able to suspend the mesalazine therapy and therefore only took levetiracetam (1,500 mg) at the time of stabilization.

## Discussion

We present the case of a patient with a schizophrenia-like disorder and, following our judgment, a (para)epileptic pathomechanism, because of the distinct epileptiform discharges without seizures and remission under the anticonvulsive treatment with topiramate and subsequently levetiracetam monotherapy.

### Reason and Potential Pathophysiology of Network Instability

The EEG alterations might be due to a polygenetic effect caused by different genes (12). The immunological mechanisms in the framework of ulcerative colitis and increased antinuclear antibody titers might have supported the network instability by mild inflammatory processes (7, 13–15). Medication might also disclose underlying polygenetic or immunological network instability (16). The local area network inhibition (LANI) hypothesis might explain the causal relationship between epileptiform EEG discharges and schizophrenia-like symptoms. Excitatory network activity, as represented by the 3 Hz polyspike wave complexes, might lead to consecutive inhibitory processes in a physiological attempt of the central nervous system to stabilize the excitatory–inhibitory equilibrium of local cerebral networks. The repetitive excitatory activity, as documented by consecutive EEGs in our patient, could have exceeded a critical threshold, leading to the successive hyperinhibition of cerebral networks. Following the LANI hypothesis, the symptoms are due to the secondary induced processes of hyperinhibition (e.g., temporal hyperinhibition might have led to auditory hallucinations or memory deficits) (2–5, 17).

### Treatment Considerations

Following the LANI hypothesis, the cognitive improvement after the addition of valproate to the neuroleptic treatment would be explained by the reduced epileptic activity and therefore the subsequent amelioration of inhibitory processes. In line with this assumption, comprehensive long-term stabilization was not achieved by several attempts of neuroleptic medication alone in spite of clear and very convincing effects of the treatment with clozapine in particular on positive symptoms. However, such a comprehensive improvement and even full remission was achieved with topiramate and later levetiracetam monotherapy. Thus, clozapine with its well-known proconvulsive properties might well have counteracted inhibitory processes, while it is at the same time most likely unable to improve causative excitatory neuronal activity. By contrast, by reducing the epileptiform activity, topiramate and levitiracetam monotherapy might have resulted in a more causal and therefore more comprehensive improvement of relevant pathophysiology. Earlier, we published a case of a (para)epileptic schizophrenia-like disorder successfully treated with valproate (4). Valproate, and likewise lamotrigine, is already established as an augmentative treatment strategy in schizophrenia (18). One might hypothesize that patients with (para)epileptic pathomechanisms will benefit significantly more from antiepileptic treatment than other subgroups. To our knowledge, this is the first published case study that describes a patient with a schizophrenia-like disorder who was successfully treated with levetiracetam. Levetiracetam is rarely used off-label in psychiatry probably because of its potential side effects, such as agitation, aggression, fear, and psychosis (2). The advantage of levetiracetam is that it can be rapidly dosed up to effective concentrations. Therefore, on a single case basis, the working hypothesis of a (para)epileptic pathomechanism could be tested quickly. In comparison, valproate effects could be due to combined γ-aminobutyric acid (GABAergic) and antiglutamatergic effects, and lamotrigine effects might be due to potential antiglutamatergic effects. However, the mechanism of levetiracetam cannot be explained by such direct transmitter effects. The effects of levetiracetam seem to be associated with the binding of the synaptic vesicle glycoprotein 2A (SV2A) (2). SV2A can be found in presynaptic membranes; it controls the calcium-dependent exocytosis of different neurotransmitters into the synaptic gap (19, 20). Therefore, it might also influence GABAergic and glutamatergic transmission (21).

### Limitations

Epileptiform discharges are found in less than 1% of healthy adults (1, 22–24) and as a rare consequence of clozapine treatment (16). Therefore, the EEG alterations could be interpreted either as an incidental finding in a patient with schizophrenia or as a clozapine side effect. However, the clinical course—with improvement under antiepileptic treatment in parallel with EEG normalization—speaks against the assumption. The pathophysiological processes might be explained by the LANI hypothesis; however, this is only an unproven, theoretical framework that needs further investigation.

### Conclusion

This case report illustrates the idea of a possible (para)epileptic pathomechanism in a patient with a schizophrenia-like disorder. Regarding diagnostic procedure, our case shows the importance of EEG examinations in typical schizophrenia-like disorders. Regarding pathophysiology, the case illustrates a potential pathogenetic role of epileptiform discharges in a subgroup of patients with schizophrenia-like symptoms. Regarding treatment, the case demonstrates that anticonvulsive medication with levetiracetam and also topiramate or valproate could be a successful treatment alternative in schizophrenia with EEG alterations.

## Ethics Statement

The patient has given her informed and written consent for this case report, including the presented images, to be published.

## Author Contributions

LTvE treated the patient. DE wrote the paper and performed the data collection. DE and LTvE performed the interpretation of the diagnostic findings and therapy effects. BF performed and interpreted the EEG analysis. NV performed and interpreted the immunological analyses. EP and D-MA reviewed the diagnostic results and contributed to the manuscript preparation. All the authors were significantly involved in the theoretical discussion and the preparation of the manuscript, and they read and approved the final version of the manuscript.

## Conflict of Interest Statement

DE, EP, and BF: none; D-MA: lecture fees from UCB Pharma; NV: advisory boards, lectures, research, or travel grants within the last 3 years: Janssen-Cilag, Roche, Novartis, AbbVie, GSK, and Medac. LTvE: lectures, work shops, or travel grants within the last 3 years: Eli Lilly, Medice, Shire, UCB, Servier, and Cyberonics.

## Footnotes

^1^http://apps.who.int/classifications/icd10/browse/2010/en#/F20-F29.

^2^www.rheumatology.org/Portals/0/Files/1997%20Update%20of%201982%20Revised.pdf.

## Abbreviations

ACR, American College of Rheumatology; ANA, antinuclear antibody; CSF, cerebrospinal fluid; EEG, electroencephalography; FIRDA, frontal intermittent rhythmic delta activity; GABA, γ-aminobutyric acid; HV, hyperventilation; ICA, independent component analyses; IRDA; intermittent rhythmic delta activity; LANI, local area network inhibition; SLE, systemic lupus erythematosus; SV2A, synaptic vesicle glycoprotein 2A.
